# The First Meta-Analysis of the M129V Single-Nucleotide Polymorphism (SNP) of the Prion Protein Gene (*PRNP*) with Sporadic Creutzfeldt–Jakob Disease

**DOI:** 10.3390/cells10113132

**Published:** 2021-11-11

**Authors:** Yong-Chan Kim, Byung-Hoon Jeong

**Affiliations:** 1 Korea Zoonosis Research Institute, Jeonbuk National University, Iksan 54531, Korea; kych@jbnu.ac.kr; 2 Department of Bioactive Material Sciences, Institute for Molecular Biology and Genetics, Jeonbuk National University, Jeonju 54896, Korea

**Keywords:** prion, *PRNP*, CJD, polymorphism, SNP, susceptibility, M129V, meta-analysis

## Abstract

Prion diseases are fatal, chronic, and incurable neurodegenerative diseases caused by pathogenic forms of prion protein (PrP^Sc^) derived from endogenous forms of prion protein (PrP^C^). Several case–control and genome-wide association studies have reported that the M129V polymorphism of the human prion protein gene (*PRNP*) is significantly associated with susceptibility to sporadic Creutzfeldt–Jakob disease (CJD). However, since some case–control studies have not shown these associations, the results remain controversial. We collected data that contain the genotype and allele frequencies of the M129V single-nucleotide polymorphism (SNP) of the *PRNP* gene and information on ethnic backgrounds from sporadic CJD patients. We performed a meta-analysis by collecting data from eligible studies to evaluate the association between the M129V SNP of the *PRNP* gene and susceptibility to sporadic CJD. We found a very strong association between the M129V SNP of the *PRNP* gene and susceptibility to sporadic CJD using a meta-analysis for the first time. We validated the eligibility of existing reports and found severe heterogeneity in some previous studies. We also found that the MM homozygote is a potent risk factor for sporadic CJD compared to the MV heterozygote in the heterozygote comparison model (MM vs. MV, odds ratio = 4.9611, 95% confidence interval: 3.4785; 7.0758, *p* < 1 × 10^−10^). To the best of our knowledge, this was the first meta-analysis assessment of the relationship between the M129V SNP of the *PRNP* gene and susceptibility to sporadic CJD.

## 1. Introduction

Prion diseases are chronic, lethal, and malignant neurodegenerative diseases caused by toxic forms of prion protein (PrP^Sc^) derived from benign prion protein (PrP^C^), which is encoded by the prion protein gene (*PRNP*) [1,2,3,4,5]. In humans, prion diseases are classified into three types: sporadic, genetic, and acquired. The most common type of human prion disease is sporadic Creutzfeldt–Jakob disease (CJD), which accounts for approximately 85% of all CJD cases. Genetic forms of human prion disease, accounting for 10–15% of all CJD cases, occur due to germline mutations of the *PRNP* gene. These forms include fatal familial insomnia (FFI) with the D178N-129M genotype; Gerstmann–Sträussler–Scheinker syndrome (GSS) with P102L, A117V, and F198S mutations; and genetic CJD, including the G114V, D178N-129V, V180I, E200K, and V210I mutations. Acquired forms of human prion disease account for less than 1% of all CJD cases, including iatrogenic CJD, kuru, and variant CJD, which are caused by consuming meat from bovine spongiform encephalopathy (BSE)-affected cattle [6,7,8,9,10]. However, the cause of sporadic CJD has not been elucidated thus far.

In previous genome-wide association studies (GWAS), the locus of the *PRNP* gene was found to be extremely related to susceptibility to sporadic CJD [11,12]. Several case–control studies using fine mapping have also identified that the nonsynonymous polymorphism at codon 129 of the *PRNP* gene is significantly associated with susceptibility to sporadic CJD [10,13,14,15,16,17,18,19,20,21,22,23,24,25]. However, the control populations used in several case–control studies were not in a Hardy–Weinberg equilibrium (HWE) and showed severe heterogeneity in the value of association [15,19,20,22,24]. In addition, the genotype and allele distributions of the M129V SNP were not shown to be related to vulnerability to sporadic CJD in the Brazilian population, and the allele distribution of the M129V single-nucleotide polymorphism (SNP) was not found to be associated with the susceptibility to sporadic CJD in several other case–control studies [13,15,18,19,23]. Furthermore, since some studies have provided information only on sporadic CJD patients, an association test was not available in those studies [23,25]. Thus, a comprehensive evaluation of the association between polymorphisms of the *PRNP* gene at codon 129 and susceptibility to sporadic CJD is needed in quality-checked and quantitatively synthesized studies.

To evaluate the association between the M129V SNP of the *PRNP* gene and susceptibility to sporadic CJD, we collected data on the genotype and allele frequencies of the M129V SNP of the *PRNP* gene and information on ethnic backgrounds from sporadic CJD patients. The information on matched-control populations was supplemented from the 1000 Genomes Project and used for an association analysis. Then, we performed a meta-analysis by collecting data from eligible studies to evaluate the association of the M129V SNP of the *PRNP* gene with susceptibility to sporadic CJD.

## 2. Materials and Methods

### 2.1. Literature Search

A literature search was performed in the PubMed database to identify studies relating to the M129V SNP of the *PRNP* gene from sporadic CJD patients. The following search terms were used: “*PRNP*”, “prion”, “CJD”, or “Creutzfeldt–Jakob disease” combined with “SNP” or “polymorphism” or “susceptibility” (the last search update was performed on 8 March 2021). We also supplemented our search by screening reference lists of all the relevant studies, including original articles and reviews. Irrelevant studies were excluded after the initial screening of titles and abstracts. Eligible studies met the following inclusion criteria: (1) relating to the association between the M129V SNP and sporadic CJD; (2) being a cohort or case–control study; (3) containing genetic information on the M129V SNP of sporadic CJD patients; (4) having full text available; and (5) being published in English. The exclusion criteria were as follows: (1) animal studies; (2) case reports; and (3) insufficient genotype data.

### 2.2. Association Analysis

We collected 13 studies that contained the genotype and allele frequencies of the M129V SNP of the *PRNP* gene in sporadic CJD patients and information on their ethnic background. Information on matched control populations, including Caucasian and East Asian populations, was obtained from the 1000 Genome Project. Comparison of the genotype and allele frequencies between sporadic CJD patients and control populations was analyzed using SAS version 9.4 (SAS Institute Inc., Cary, NC, USA). Statistical significance was measured by *p*-values obtained using the χ^2^ test and Fisher’s exact test. The HWE test was performed using Haploview version 4.2 (Broad Institute, Cambridge, MA, USA).

### 2.3. Meta-Analysis

The strength of the association between the M129V SNP of the *PRNP* gene and susceptibility to sporadic CJD was estimated in a meta-analysis. The pooled odds ratios with 95% confidence intervals were calculated based on additive (M vs. V), recessive (MM vs. MV + VV), dominant (MM + MV vs. VV), and over-dominant (MV vs. MM + VV) genetic models and homozygote (MM vs. VV) and heterozygote (MM vs. MV and MV vs. VV) comparisons. Heterogeneity was evaluated by the *p*-value and I^2^ value. Fixed and random effect models were selected to calculate the pooled odds ratios according to the value of the I^2^ test. Publication bias was evaluated using Egger’s weighted regression methods. A meta-analysis was conducted using the meta package of the R program (https://www.r-project.org/ (accessed on 14 August 2021)).

## 3. Results

### 3.1. Investigation of the Association between the M129V SNP of the PRNP Gene and Susceptibility to Sporadic CJD in Each Group

We searched 278 research articles using the search terms “*PRNP*”, “prion”, “CJD”, or “Creutzfeldt–Jakob disease” combined with “SNP” or “polymorphism” or “susceptibility” (the last search update was performed on 8 March 2021) in PubMed. After excluding duplicate and irrelevant articles, a total of 13 relevant studies were extracted from the databases based on our inclusion and exclusion criteria.

To identify an association between the M129V SNP of the *PRNP* gene and susceptibility to sporadic CJD, we performed an association analysis between sporadic CJD patients and matched control populations, including Caucasian and East Asian populations obtained from the 1000 Genomes Project. Except for two groups, Salvatore et al. 1994 and Croes et al. 2004, the genotype frequencies of the control population of all groups tested were in HWE. Except for one group, Martins 2007, the genotype frequencies of M129V of the *PRNP* gene exhibited a strong association (*p* < 0.05) with susceptibility to sporadic CJD in all groups tested (Table 1). Except for five groups—Palmer et al. 1991, Croes et al. 2004, Martins et al. 2007, Bishop et al. 2009, and Kobayashi et al. 2015—the allele frequencies of the M129V SNP of the *PRNP* gene also showed a strong association (*p* < 0.05) with the vulnerability of sporadic CJD in all groups tested.

### 3.2. Evaluation of the Association between the M129V SNP of the PRNP Gene and Susceptibility to Sporadic CJD by Meta-Analysis

First, we performed a meta-analysis with all 13 groups tested and found severe heterogeneity and publication bias induced by Doh-ura et al. 1991, Salvatore et al. 1994, Croes et al. 2004, Jeong et al. 2005 and Bishop et al. 2009 (data not shown). Except for the five studies listed, a total of eight studies showing the association between the M129V SNP and susceptibility to sporadic CJD were included in this meta-analysis (Table 1, shaded blocks). In total, 3290 sporadic CJD patients and 3415 controls were included in the meta-analysis. The pooled odds ratios with 95% confidence intervals were calculated based on additive, recessive, dominant, and over-dominant genetic models and homozygote and heterozygote comparisons. Heterogeneity among the collected studies was tested using the *p*-value and I^2^ value (Table 2). According to the I^2^ value of these studies with no heterogeneity, we used fixed (<50%) and random (>50%) models for the meta-analysis.

**Figure 1:**
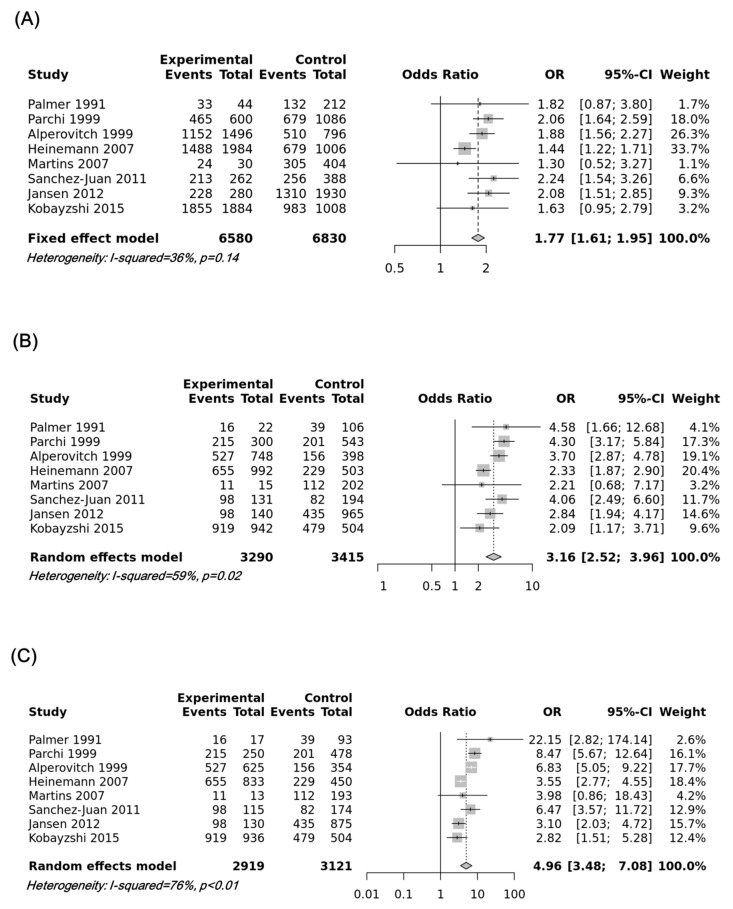
(**A**) Forest plot of the association between the M129V single-nucleotide polymorphism (SNP) of the *PRNP* gene and susceptibility to sporadic Creutzfeldt–Jakob disease (CJD) in the additive model (M vs. V). (**B**) The forest plot for the association between the M129V SNP of the *PRNP* gene and susceptibility to sporadic CJD in the recessive model (MM vs. MV + VV). (**C**) The forest plot for the association between the M129V SNP of the *PRNP* gene and susceptibility to sporadic CJD in the heterozygote comparison (MM vs. MV).

Our data revealed an association between the risk of sporadic CJD and the M129V SNP of the *PRNP* gene in the additive model (odds ratio = 1.7698, 95% confidence interval: 1.6068; 1.9494, *p* < 0.0001), recessive model (odds ratio = 3.1556, 95% confidence interval: 2.5163; 3.9574, *p* < 0.0001, dominant model (odds ratio = 0.6722, 95% confidence interval: 0.5569; 0.8114, *p* < 0.001), over-dominant model (odds ratio = 0.2088, 95% confidence interval: 0.1483; 0.2939, *p* < 0.0001), and heterozygote comparisons (MM vs. MV, odds ratio = 4.9611, 95% confidence interval: 3.4785; 7.0758, *p* < 0.0001; MV vs. VV, odds ratio = 0.2256, 95% confidence interval: 0.1810; 0.2811, *p* < 0.0001). However, no significant association was found in the homozygote comparison (odds ratio = 1.1906, confidence interval: 0.9788; 1.4483, *p* = 0.5666). To examine potential publication bias, Egger’s tests were performed, and publication bias was not observed in this meta-analysis (*p* > 0.1). The details of the outcomes are shown in Table 2. The most significant association was observed in the heterozygote comparison (MM vs. MV), followed by the recessive model and additive model. Forest plots of the heterozygote comparison (MM vs. MV), recessive model, and additive model are drawn in Figure 1.

## 4. Discussion

In the present study, we evaluated the association between the M129V SNP of the *PRNP* gene and susceptibility to sporadic CJD using a meta-analysis. To do so, we validated the eligibility of each study and identified that several studies were not suitable due to HWE violation and significant heterogeneity (Table 1). We performed a meta-analysis on qualifying studies and identified a significant association between the M129V SNP of the *PRNP* gene and susceptibility to sporadic CJD in all genetic models, except for the homozygote comparison (Table 2). Notably, the strongest association was found in the heterozygote comparison (MM vs. MV). In previous studies, heterozygosity at codon 129 of the human *PRNP* gene was found to be related to the resistance of human prion diseases. Sporadic CJD patients and kuru with the MV genotype at codon 129 of the human *PRNP* gene showed later disease onset and longer incubation time [28,29,30]. In addition, human PrP transgenic mice with the MV genotype at codon 129 of the human *PRNP* gene showed a prolonged incubation period [27,31,32]. These studies indicated that the heterozygote comparison model showed a correlation with the pathomechanism of prion diseases according to the genotypes of *PRNP* polymorphisms at codon 129.

After the validation of previous case–control studies, we found that the remaining studies were primarily composed of Caucasian populations (75%, Table 1). Since the number of case–control studies in East Asia and South America is not sufficient to evaluate the susceptibility to sporadic CJD by meta-analysis, further case–control studies are highly desirable in various countries and ethnic backgrounds. In addition, two studies in the Korean and Japanese populations showed severe heterogeneity and were excluded from the current meta-analysis (Table 1). The data in the Japanese population [22] and the Korean population [24] showed a low odds ratio (0.37) and an extremely high odds ratio (17.78) in the heterozygote comparison model (MM vs. MV, data not shown), respectively. However, there was no remarkable difference in the incidence of sporadic CJD between the two countries. Thus, these results indicate that another genetically susceptible factor may be involved in susceptibility to sporadic CJD. Previous studies have shown that codon 129 polymorphism of the human *PRNP* gene in the Asian population has rarely been found in sporadic CJD patients, and the heterozygote of this SNP has been considered to have a resistant effect. Thus, the further estimation of the effect of the M129V SNP combined with the genotype and allele frequencies of the E219K SNP on susceptibility to sporadic CJD is needed in future studies. In addition, to date, since GWAS has not been conducted in the Asian population thus far, a large-scale GWAS consisting of a coalition of Asian countries may be necessary in order to discover novel genetic factors related to susceptibility to sporadic CJD in the Asian population. Furthermore, because the CJD patients used in this study were not subdivided based on the PrP^Sc^ banding pattern into subtypes, including MM1, MM2, MV1, MV2, VV1, and VV2, a further analysis conducted according to these subtypes would be highly desirable in the future. In addition, since the present meta-analysis was performed with a relatively small number of a total of 13 studies, a further analysis using a larger number of studies would be highly desirable in the future.

## 5. Conclusions

In conclusion, we identified a strong association between the M129V SNP of the *PRNP* gene and susceptibility to sporadic CJD using a meta-analysis for the first time. We validated the eligibility of previous case–control studies and found severe heterogeneity in some previous case–control studies. In addition, we found that the heterozygote comparison model (MM vs. MV) showed the highest risk of susceptibility to sporadic CJD in the meta-analysis. MM homozygote is an especially potent risk factor of sporadic CJD compared to MV heterozygote. To the best of our knowledge, this was the first meta-analysis assessment of the association between the M129V SNP of the *PRNP* gene and susceptibility to sporadic CJD.

## Figures and Tables

**Table 1:** Comparison of the genotype and allele frequencies of M129V in the prion protein gene (*PRNP*) between sporadic Creutzfeldt–Jakob disease (sCJD) patients and matched control populations (CTL) in previous studies.

Study	Year	Ethnicity		Total, n	Genotype Frequencies, n			*p*-Value	Allele Frequencies, n		*p*-Value	HWE	Ref
					MM	MV	VV		M	V			
Doh-ura et al.	1991	East Asian	sCJD	21	16	4	1	<0.01	36	6	<0.01	0.3085	[22]
			CTL	179	164	15	0		343	15		0.6153	[22]
Palmer et al.	1991	Caucasian	sCJD	22	16	1	5	<0.001	33	11	0.1082	<0.001	[23]
			CTL	106	39	54	13		132	80		0.5624	[21]
Salvatore et al.	1994	Caucasian	sCJD	31	25	5	1	<0.001	55	7	<0.001	0.2780	[20]
			CTL	186	84	27	75		195	177		<0.0001	[20]
Parchi et al.	1999	Caucasian	sCJD	300	215	35	50	0.0001	465	135	<0.001	0.0001	[14]
			CTL	544	201	277	65		679	407		0.1694	[14]
Alperovitch et al.	1999	Caucasian	sCJD	748	527	98	123	0.0001	1152	344	<0.001	<0.0001	[10]
			CTL	398	156	198	44		510	286		0.3513	[10]
Croes et al.	2004	Caucasian	sCJD	42	24	12	6	0.0120	60	24	0.8990	0.0519	[19]
			CTL	241	112	117	12		341	141		0.0474	[19]
Jeong et al.	2005	East Asian	sCJD	150	150	0	0	0.0116	300	0	<0.001	N.A.	[24]
			CTL	529	499	29	1		1027	31		0.5624	[24]
Heinemann et al.	2007	Caucasian	sCJD	992	655	178	159	<0.0001	1488	496	<0.001	<0.0001	[17]
			CTL	503	229	221	53		679	327		0.9764	1000 Genome Project
Martins et al.	2007	South American	sCJD	15	11	2	2	0.0616	24	6	0.5782	0.0238	[18]
			CTL	202	112	81	9		305	99		0.5068	[18]
Bishop et al.	2009	Caucasian	sCJD	309	184	66	59	<0.0001	434	184	0.8154	<0.0001	[15]
			CTL	192	90	87	15		267	117		0.5624	[15]
Sanchez-Juan et al.	2011	Caucasian	sCJD	131	98	17	16	<0.0001	213	49	<0.0001	<0.0001	[16]
			CTL	194	82	92	20		256	132		0.5624	[16]
Jansen et al.	2012	Caucasian	sCJD	140	98	32	10	<0.0001	228	52	<0.0001	0.0038	[25]
			CTL	965	435	440	90		1310	620		0.4082	[26]
Kobayashi et al.	2015	East Asian	sCJD	942	919	17	6	<0.001	1855	29	0.0748	<0.0001	[27]
			CTL	504	479	25	0		983	25		0.6153	1000 Genome Project

The numbers in each column of “Allele frequencies” indicate the number of alleles. The number of “Total, n” indicates the number of people. Shaded blocks indicate the studies used in the meta-analysis. HWE: Hardy–Weinberg equilibrium; N.A.: not available.

**Table 2:** Meta-analysis of the association between M129V of the prion protein gene (*PRNP*) and susceptibility to sporadic Creutzfeldt–Jakob disease (CJD).

Genetic Model	Association test			Heterogeneity			Publication Bias
	Odds Ratio	95% Confidence Interval	*p*-Value	Model	*p*-Value	I^2^	Egger’s Test *p*-Value
Additive model (M vs. V)	1.7698	[1.6068; 1.9494]	< 0.0001	Fixed	0.14	0.36	0.6318
Recessive model (MM vs. MV + VV)	3.1556	[2.5163; 3.9574]	< 0.0001	Random	0.02	0.59	0.8076
Dominant model (MM + MV vs. VV)	0.6722	[0.5569; 0.8114]	< 0.001	Fixed	0.43	0.00	0.6068
Over-dominant model (MV vs. MM + VV)	0.2088	[0.1483; 0.2939]	< 0.0001	Random	0.00	0.75	0.6885
MM vs. VV	1.1906	[0.9788; 1.4483]	0.5666	Fixed	0.31	0.15	0.5145
MM vs. MV	4.9611	[3.4785; 7.0758]	< 0.0001	Random	0.00	0.76	0.6721
MV vs. VV	0.2256	[0.1810; 0.2811]	< 0.0001	Fixed	0.04	0.52	0.5189

## Data Availability

Data are available on reasonable request. Requests may be made to bhjeong@jbnu.ac.kr.
